# A Case Pulmonary Tuberculosis Treated by Artificial Phemothorax

**Published:** 1910-10

**Authors:** Mary E. Lapham

**Affiliations:** Highlands Camp Sanatorium, Highlands, N. C.


### A CASE PULMONARY TUBERCULOSIS TREATED BY ARTIFICIAL PHEMOTHORAX.

By Mary E. Lapham, M. D.

Highlands Camp Sanatorium, Highlands, N. C.

The following case is interesting because it illustrates the disastrous effects that pelvic influences may exert upon an otherwise favorable case of unilateral pulmonary tuberculosis.

H. M. School girl, 17, admitted to Highlands Camp Sanatorium July 2, 1909.

History. In 1906 began feeling weak and tired and had a slight cough; no appetite and loss of weight. In May, 1908, menstruation ceased. December, 1908, mother became seriously alarmed on account of extreme weakness and violent paroxysms of coughing. During Christmas week 1908, efforts to menstruate were made. On January 15, 1909, a severe paroxysm of coughing was followed by a profuse hemorrhage, saturating several handkerchiefs, cloths, etc. January 16, second hemorrhage, not quite so severe. March 16, third hemorrhage. June 4th and 5th two more hemorrhages, followed by spitting of blood for days.

Family History negative.

Status Presens. Frail, delicate build, with high color in the cheeks and deep dark rings under the eyes. H6. 60 R. B. C. 3,-000,000, Pulse 114, temperature 96.8 to 99. Sputum positive with quantities alveolar cells, sometimes occurring in patches, Breathing quick and shallow, 26. Dyspnoea on exertion, great aversion to food, and very little strength. Right lung, normal resonance and vesicular breath sounds. Left lung, in front, slightly relative dullness to the second rib, slight dullness to the fifth rib. Supra clavicular space, rough, suppresed inspiration, rough exaggerated exspiration. First interscostal space the same. Second intercostal space very uneven, supprssed inspiration. In the fourth and fifth interspaces large wet rales after coughing. Behind—slightly relative dullness to the spine of the scapula; slight dullness to the angle; suppressed, uneven inspiration to the spine. After coughing large wet rales to the angle of the scapula. The whole of the left upper lobe was involved and the upper two-thirds of the lower.

